# Age-related changes in layer II immature neurons of the murine piriform cortex

**DOI:** 10.3389/fncel.2023.1205173

**Published:** 2023-07-28

**Authors:** Marco Ghibaudi, Nicole Marchetti, Elena Vergnano, Chiara La Rosa, Bruno Benedetti, Sebastien Couillard-Despres, Stefano Farioli-Vecchioli, Luca Bonfanti

**Affiliations:** ^1^Neuroscience Institute Cavalieri Ottolenghi (NICO), Orbassano, Italy; ^2^Department of Veterinary Sciences, University of Turin, Turin, Italy; ^3^Institute of Biochemistry and Cell Biology, National Research Council, Rome, Italy; ^4^Institute of Experimental Neuroregeneration, Paracelsus Medical University, Salzburg, Austria; ^5^Spinal Cord Injury and Tissue Regeneration Center Salzburg (SCI-TReCS), Salzburg, Austria; ^6^Austrian Cluster for Tissue Regeneration, Vienna, Austria

**Keywords:** brain plasticity, cerebral cortex, piriform cortex, neurogenesis, mice, aging

## Abstract

The recent identification of a population of non-newly born, prenatally generated “immature” neurons in the layer II of the piriform cortex (cortical immature neurons, cINs), raises questions concerning their maintenance or depletion through the lifespan. Most forms of brain structural plasticity progressively decline with age, a feature that is particularly prominent in adult neurogenesis, due to stem cell depletion. By contrast, the entire population of the cINs is produced during embryogenesis. Then these cells simply retain immaturity in postnatal and adult stages, until they “awake” to complete their maturation and ultimately integrate into neural circuits. Hence, the question remains open whether the cINs, which are not dependent on stem cell division, might follow a similar pattern of age-related reduction, or in alternative, might leave a reservoir of young, undifferentiated cells in the adult and aging brain. Here, the number and features of cINs were analyzed in the mouse piriform cortex from postnatal to advanced ages, by using immunocytochemistry for the cytoskeletal marker doublecortin. The abundance and stage of maturation of cINs, along with the expression of other markers of maturity/immaturity were investigated. Despite a marked decrease in this neuronal population during juvenile stages, reminiscent of that observed in hippocampal neurogenesis, a small amount of highly immature cINs persisted up to advanced ages. Overall, albeit reducing in number with increasing age, we report that the cINs are present through the entire animal lifespan.

## Introduction

The mammalian brain is a highly complex machine needing stability (Frotscher, 1992), and hardly capable of renewing its neuronal elements, especially in mammals (Bonfanti, 2011, 2013; Semënov, 2021). These features are less strict during youth, thanks to the existence of remarkable brain plasticity, yet they can become a problem with aging (Ben Abdallah et al., 2010; Grady, 2012; Snyder, 2019). Brain structural plasticity can occur in different forms, from synaptic changes (formation/elimination of synaptic contacts) to the genesis of new neurons, referred to as adult neurogenesis (Bond et al., 2015; Lim and Alvarez-Buylla, 2016; La Rosa et al., 2020a; Bonfanti and Charvet, 2021). It is well known that some forms of plasticity, especially the striking structural changes involving persistent neurogenesis, progressively decline as the animal age progresses (Ben Abdallah et al., 2010; Sanai et al., 2011; Snyder, 2019; Bonfanti and Charvet, 2021; Duque et al., 2021; Bond et al., 2022). In stem cell-driven adult neurogenesis, the decrease is due to stem cell depletion and/or transition to a quiescent state. In the last few years, a counterintuitive example of “neurogenesis without division” has been shown to exist (Gómez-Climent et al., 2008; Klempin et al., 2011; Bonfanti and Nacher, 2012; König et al., 2016; La Rosa et al., 2020a; Bonfanti and Seki, 2021). It consists of prenatally generated neurons (Gómez-Climent et al., 2008; Piumatti et al., 2018) which stop their maturation for long periods, being capable to restart it during adulthood to eventually integrate into neural circuits (Rotheneichner et al., 2018; Benedetti et al., 2020). These immature “standby” or “dormant” neurons are here referred to as cortical immature neurons (cINs). cINs are present in the cerebral cortex, a brain region not endowed with stem cell-driven neurogenesis and characterized by high cognitive functions. The recent demonstration that cINs are more abundant and more widely distributed in large-brained, gyrencephalic mammals with respect to rodents (La Rosa et al., 2020b), suggests that they represent an evolutionary choice to grant new neurons as a sort of brain reserve in animal species endowed with high computational/cognitive capabilities linked to expanded neocortex (Palazzo et al., 2018; La Rosa et al., 2019, 2020b; Benedetti and Couillard-Despres, 2022).

Although the currently available information is still fragmentary, it appears that the number of layer II cINs varies across animal ages (Xiong et al., 2008; Cai et al., 2009; Piumatti et al., 2018; Rotheneichner et al., 2018; La Rosa et al., 2020b; Ai et al., 2021; Li et al., 2023). A general reduction in the extent of brain structural plasticity is known to affect all species, from non-mammalian vertebrates to mammals (Bonfanti, 2011), likely linked to lifespan and to a role in refinement of neural circuitries during brain growth and maturation (Luo et al., 2006; Ben Abdallah et al., 2010; Encinas et al., 2011; Sanai et al., 2011; Semënov, 2019, 2021; Snyder, 2019; Bonfanti and Charvet, 2021; Bond et al., 2022). For mammalian adult neurogenesis, such a reduction has been carefully described (Luo et al., 2006; Ben Abdallah et al., 2010) and is considered to be associated with a decrease in cell division (Semënov, 2021), as a consequence of progressive exhaustion/depletion/quiescence of neural stem cells (Encinas et al., 2011; Urbán et al., 2019).

**FIGURE 1:**
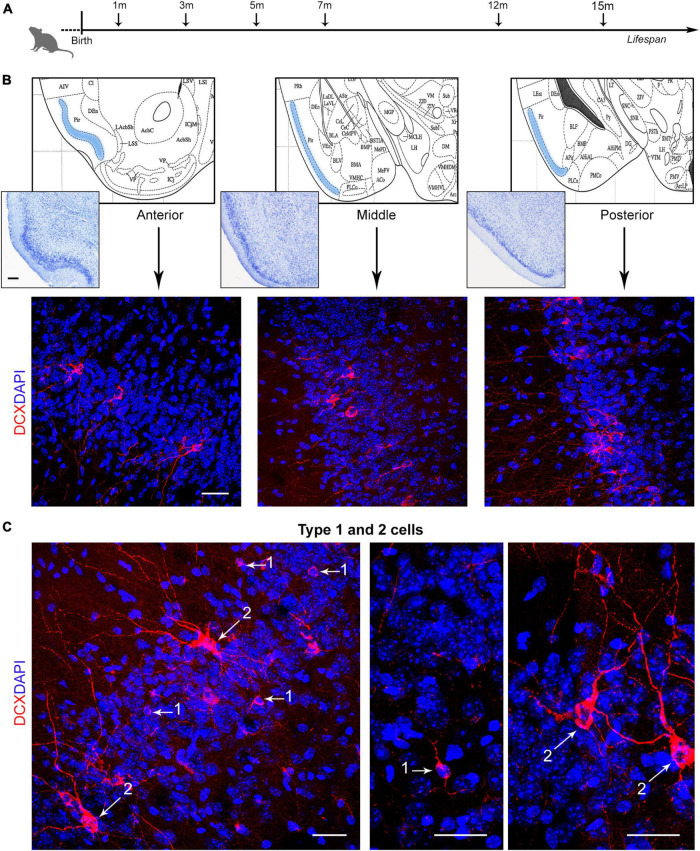
Analysis of layer II cortical immature neurons (cINs) of the mouse piriform cortex at different ages. **(A)** Schematic representation of the age groups investigated in the present study. [**(B)**, top] Three anterior-posterior levels of the piriform cortex were considered (represented from coronal views of the Paxinos atlas of the mouse brain; Paxinos and Franklin, 2019): anterior, Interaural 4.98–Bregma 1.18; middle, 2.22–1.58; posterior, 1.26–2.54. [**(B)**, bottom] Representative confocal images of each piriform cortex region (3 month-old mouse), stained for doublecortin (DCX, red) and counterstained with DAPI. **(C)** Confocal images of DCX^+^ type 1 and type 2 cells, representing, respectively small, unipolar/bipolar neuronal precursors and large, ramified “complex” cells. Scale bars: 200 μm (histology); 30 μm (immunofluorescence).

**FIGURE 2:**
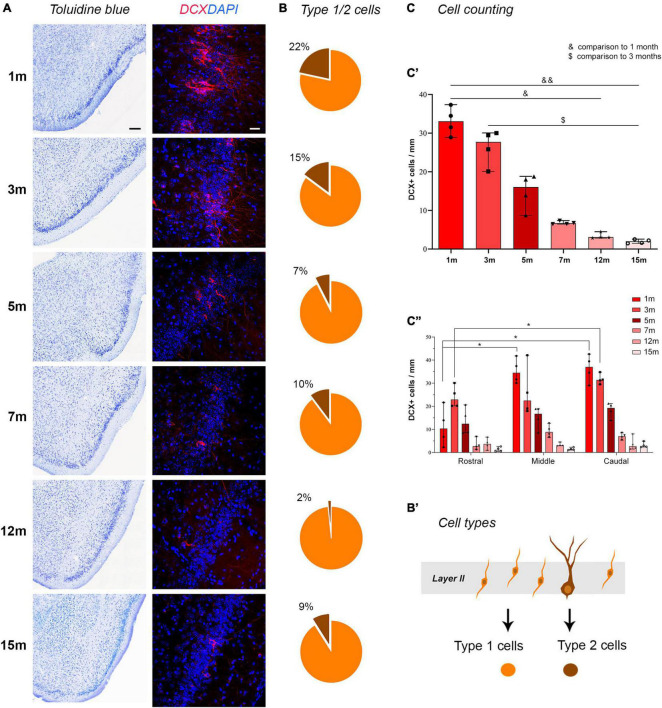
Detection and counting of DCX^+^ cells in the piriform cortex at different ages. **(A)** representative photographs from histological staining of the piriform cortex (caudal domain) and confocal images of DCX staining at all ages considered. Note the age-dependent progressive reduction in cINs. **(B)** Pie charts indicating the percentages of type 1 (yellow) and type 2 cells (brown); note the progressive decrease of type 2 “complex” cells with respect to the type 1, highly immature cells. **(B’)** Schematic representation of the type 1 (yellow) and type 2 cells (brown) corresponding to different morphologies and different maturational stages of the cINs. **(C)** Results of the cell counting carried out on ImageJ software; **(C’)** total DCX^+^ cell counting; **(C”)** counting within the rostral, middle, and caudal domain of the piriform cortex. Scale bars: 200 μm (histology); 30 μm (immunofluorescence).

The question remains open whether also the cINs, which are not dependent on stem cell division, might follow a similar pattern of reduction or whether they might be considered as a reservoir of young, undifferentiated neurons in the adult/aging brain (König et al., 2016; La Rosa et al., 2019; Benedetti and Couillard-Despres, 2022). To answer this question, we investigated the amount of DCX^+^ cINs in the mouse paleocortex at six different ages, from postnatal month 1 to the old age of 15 months (Figure 1A), at three anterior-posterior levels of the piriform cortex (Figure 1B). Cell density (linear density: number of cells/mm in the cortical layer II perimeter) and percentage of type 1 (small, unipolar/bipolar, highly immature neuronal precursors) and type 2 cINs (large, ramified, less immature “complex” cells; see Figure 2B’) were considered (Figure 1C). Furthermore, we studied the coexpression of DCX with other markers for maturity/immaturity, such as the anti-adhesive form of the Neural Cell Adhesion Molecule N-CAM (PSA-NCAM; Bonfanti, 2006), whose expression is known to maintain immaturity in the piriform cortex cINs (Coviello et al., 2021), and NeuN, a marker of post-mitotic neurons starting differentiation (Mullen et al., 1992), in search for possible variation at the different ages.

## Materials and methods

### Animals and tissue processing

Animal experiments were performed in agreement with the “Directive 2010/63/EU of the European Parliament and of the Council of 22 September 2010 on the protection of animals used for scientific purposes,” according to protocol number 785_2019PR, following the Institutional guidelines of IBBC/CNR and the approval of the Ethical Committee. Mice C57/BL6 (Charles River Laboratories, RRID:MGI:3696370) were housed in cages with corncob bedding in conventional animal facility. All animals were maintained under standard laboratory conditions with an artificial 12 h light/dark cycle (lights on at 7:00 a.m. and off at 7:00 p.m.), temperature controller (22 ± 2°C) and humidity maintenance (55 ± 5%). Mice took a standard diet with access to food and water *ad libitum*. For our experiments, only male mice were used.

Animals aged 1, 3, 5, 7, 12, 15 months (4 animals for each age; Figure 1A) were deeply anesthetized and perfused with a 4% solution of paraformaldehyde (PFA) dissolved in 0.01 M PBS. After cardiac perfusion the brains were isolated, post-fixed overnight in PFA at 4°C and equilibrated in 30% sucrose. Brains were embedded in Tissue-Tek OCT (Sakura, Alphen an den Rijn, The Netherlands), cut by cryostat at −25°C in 40 μm thick coronal, serial free-floating sections.

### Immunofluorescence for DCX, polysialylated neural cell adhesion molecule (PSA-NCAM), NeuN, and NG2

The entire anterior-to-posterior length of the piriform cortex, easily identifiable in toluidine blue-stained sections (Figure 1B), extended for approximately 5 mm at all ages; 120 coronal sections (40 μm thick) were considered in each animal (4.8 mm piriform cortex length; see Supplementary Table 1). Three different series of slices were collected: rostral sections (approximately from Bregma +2.46 to Bregma −0.34, Paxinos and Franklin, 2019), middle sections (approximately from Bregma −0.46 to Bregma −2.18), and caudal sections (approximately from Bregma −2.30 to Bregma −2.80; Figure 1B). For immunofluorescence analysis, sections were then stained by using fluorescent methods. After permeabilization with 0.3% Triton X-100 in PBS, the sections were incubated with 3% normal donkey serum in PBS for 16–18 h with primary antibodies: 1:800 goat polyclonal antibody against doublecortin (DCX) (Santa Cruz Biotechnology, Inc. Cat# sc-8066); 1:700 mouse anti-PSA-NCAM (Millipore, Billerica, MA - MAB5324), 1:300 mouse anti-NeuN (1:300, Millipore, Billerica, MA–MAB377), and 1:200 rabbit anti-NG2 (Neuron Glia antigen 2; Millipore, Billerica, MA–AB5320). Secondary antibody used to visualize the antigen were 1:200 donkey anti-goat Cy2-conjugated (Jackson ImmunoResearch Cat# 705-225-147), Alexa 647-conjugated anti-mouse (1:400; Jackson ImmunoResearch, West Grove, PA - 715-605-151), and Alexa 488-conjugated anti-rabbit (1:400; Jackson ImmunoResearch, West Grove, PA - 711-545-152). Nuclei were observed incubating sections with 4′,6-diamidino-2-phenylindole (DAPI, 1:1000, KPL, Gaithersburg, MD, USA) and mounted with MOWIOL 4–88 (Calbiochem, La jolla, CA, USA).

### Image processing and cell quantification

Images were collected using a Nikon Eclipse 90i confocal microscope (Nikon, Melville, NY, USA). All images were processed using Adobe Photoshop CS4 (Adobe Systems, San Jose, CA, USA) and ImageJ version 1.53t (Wayne Rasband, Research Services Branch, National Institute of Mental Health, Bethesda, MD, USA).

DCX^+^ cell quantification was performed on three immunofluorescence-stained sections/animal (one/each series–rostral, middle, and caudal–located in the middle of the series; 12 sections/age; total: 72 sections), in which 40× confocal fields were acquired in order to represent the entire ventral-to-dorsal length of the piriform cortex in each section (4–8 confocal fields/section, depending on the length and orientation of the piriform cortex, see Supplementary Figure 1; total number of confocal fields analyzed: 419). DCX^+^ cell counting was performed directly by an experienced operator, using “Cell Counter” Plugin of ImageJ software. In each section, confocal field (objective: 40×; corresponding to 318,215 μm × 318,215 μm) were acquired along the layer II of the paleocortex (see above). Then, the total perimeter of paleocortical layer II was traced using the “Straight line” tool of ImageJ (Supplementary Figure 1C) and all DCX^+^ cells along its length were counted in each confocal acquisition (linear density = number of cells/mm). In the same sections, the morphology of cINs was evaluated and the number of type 1 and type 2 cells (identified on the basis of their cell soma size: <9, type 1 cells; ≥9, type 2 cells) was counted using different markers selected from the Cell Counter toolbar in ImageJ software. The cell soma size (diameter) was obtained by evaluating the width orthogonal to main axis. Cells cut on the superior surface of the stack were not considered, to avoid overcounting.

Counting of DCX/PSA-NCAM, DCX/NeuN, and DCX/NG2 double staining was performed on the three-month-old and the fifteen-month-old animals. Three 40× confocal fields were considered along the ventral-to-dorsal extension of the piriform cortex, in each of two coronal sections/animal (corresponding to middle and caudal regions, which are the most enriched in DCX^+^ cells); a total of 353 (DCX/PSA-NCAM) and 252 (DCX/NeuN) coexpressing cells were counted. Similarly, counting of DCX and NG2 possible coexpression was performed as for the above-mentioned markers (on three 40× confocal fields along the ventral-to-dorsal extension of the piriform cortex, in each of two coronal sections/animal corresponding to middle and caudal regions); a total of 215 DCX^+^ cells were counted.

### Statistical analysis

All graphs and statistical analyses were performed using GraphPad Prism Software (San Diego California, USA) using different non-parametric tests: Mann-Whitney test, Kruskal-Wallis test with Dunn’s multiple comparison post-test and Two-way ANOVA with Bonferroni *post-hoc* test. *p* < 0.05 was considered as statistically significant. Median was used as a central measure.

## Results

### Immunocytochemical detection and morphology of DCX^+^ cells

**FIGURE 3:**
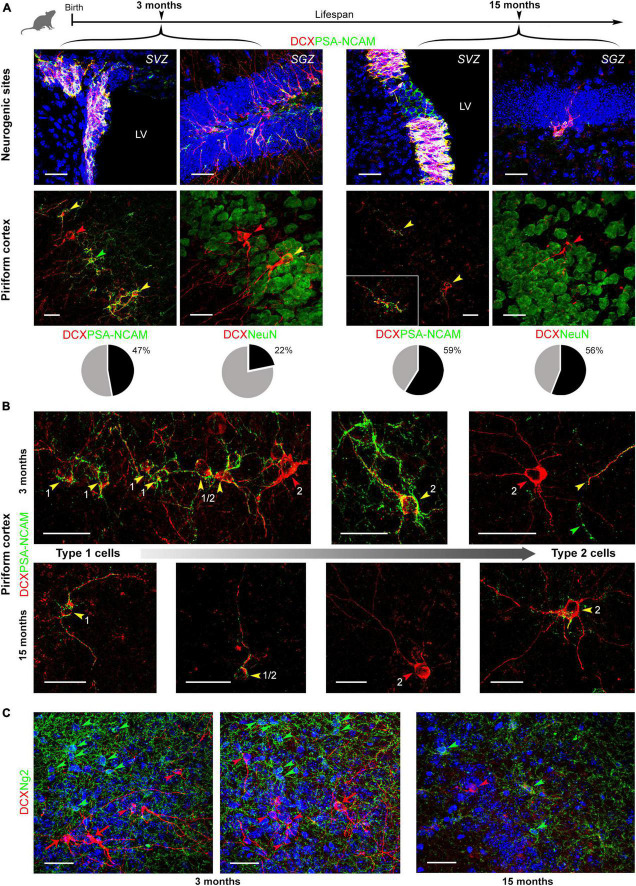
Coexpression of DCX and markers for maturity/immaturity in cINs at different ages (3 and 15 months). **(A)** Coexpression of DCX and PSA-NCAM (top, in neurogenic sites; bottom left, in piriform cortex), and DCX and NeuN (bottom right, in piriform cortex) in young and old mice. Below each double staining image in the piriform cortex, pie charts indicate the percentage of marker coexpression (gray, single-stained DCX^+^ cells; black, double-stained cells, with PSA-NCAM or NeuN, respectively). **(B)** Coexpression of DCX and PSA-NCAM in cells with different morphology reminiscent of different degrees of maturation (the smaller, unipolar/bipolar type 1 and larger, ramified type 2 cells represent the extremes). Arrowheads: red, DCX^+^ cells; green, PSA-NCAM^+^ cells; yellow, cells coexpressing the two markers. Overall, all types of cells and combinations of staining are detectable at different ages, and in different maturational stages of the cells. The coexpression of markers for immaturity (DCX and PSA-NCAM) in cell soma and processes is frequent in type 1, small cells, even at 15 months (see higher magnification in the inset). Features linked to maturity (DCX staining without PSA-NCAM or DCX/NeuN coexpression are detectable in type 2, large cells). **(C)** Double staining with anti-DCX and anti-NG2 antibodies. No coexpression of the two markers was ever found, neither in young (3-months-old) nor in old (15-months-old) animals. Green arrowheads, NG2^+^ cells; red arrowheads, DCX^+^ type 1 neurons; red arrows, DCX^+^ type 2 neurons. Scale bars: 30 μm.

Immunocytochemistry for DCX was carried out by using one of the best performing antibodies for this antigen in mice (Ghibaudi et al., 2023). As expected, different populations of DCX^+^ cells were detectable at typical locations previously described in the mouse brain, including the subventricular zone of the lateral ventricles (SVZ; Brown et al., 2003; see below and Figure 3B), the dentate gyrus of the hippocampus (subgranular zone, SGZ; Brown et al., 2003; see below and Figure 3B), and the layer II of the piriform cortex (Nacher et al., 2001; Luzzati et al., 2009; Bonfanti and Nacher, 2012; Figure 1). In the piriform cortex, two morphological types of DCX^+^ cells were detectable in layer II at the limit with layer III, as previously described (Piumatti et al., 2018; La Rosa et al., 2020b): type 1 cells, characterized by a small cell soma (diameter 5–8 μm) and very simple cell process ramification (unipolar or bipolar), and type 2 cells, with larger cell soma (diameter 8–18 μm) and complex apical dendrites (Figures 1C, 2B’). Type 1 cells, usually far more abundant than type 2 (Piumatti et al., 2018; La Rosa et al., 2020b), are known to be highly immature elements, while type 2 cells do represent more mature and morphologically more complex forms (Rotheneichner et al., 2018; Benedetti et al., 2020; Benedetti and Couillard-Despres, 2022).

### Counting of total DCX^+^ cells, and type 1/type 2 cells, in the piriform cortex

Total counting of layer II DCX^+^ cells in the whole piriform cortex, and selective counting in each anterior-middle-posterior part were performed on confocal images using ImageJ software (Figure 2C). Linear density was used considering that cINs are arranged in a monolayer-like row within the piriform cortex (see La Rosa et al., 2020b). Animals of six different adult ages were analyzed and a strong decrease in the linear density was observed comparing 1-month-old to 12- and 15-months-old mice (non-parametric Mann Whitney test, *p < 0.05; **p < 0.01, Figure 2C’) and comparing 3-months-old animals to 15-months-old ones (non-parametric Mann Whitney test, *p* < 0.05). Considering the percentage of decrease of DCX^+^ cells in piriform cortex layer II starting from 1-month-old animals, a dramatic decrease was evident between 5 and 7-months (see Table 1). Then, we calculated the mean value of total DCX^+^ cells in the ventral-to-dorsal extension of the piriform cortex in each section (average value considering rostral, middle, and caudal regions), and multiplied this value for the number of sections of the whole anterior-to-posterior extension (120), to estimate the total number of DCX^+^ cells/hemisphere at each age. Such amount ranged approximately from 20,000 cells at 1–3 months to 1,700 cells at 15 months (Supplementary Table 1).

**TABLE 1:** Percentages of DCX^+^ cells age-related reduction of cortical immature neurons (present work; Figure 4A) and hippocampal adult neurogenesis (Ben Abdallah et al., 2010; see Figure 4B).

Age comparison	Piriform cortex	Dentate gyrus
1 m vs. 2 m	–	−50%
1 m vs. 3 m	**−19%**	**−97%**
1 m vs. 4 m	–	−261%
1 m vs. 5 m	**−106%**	**−374%**
1 m vs. 7 m	**−394%**	**−1094%**
1 m vs. 9 m	–	−1568%
1 m vs. 12 m	−972%	–
1 m vs. 15 m	−1560%	–

m, months; bold, comparable ages.

To evaluate whether the distribution of DCX^+^ immature neurons might be heterogeneous through the anterior-posterior extension of the piriform cortex, the linear density obtained in three regions (anterior, middle, posterior; Figure 1B) was considered. Two-way ANOVA with Bonferroni *post-hoc* tests found no big differences among these regions at all the ages considered, apart from a slightly higher amount in middle and caudal regions with respect to rostral (Figure 2C”).

Type 1/type 2 abundance (expressed in percentage) is represented in pie charts in Figure 2B. A decrease of large type 2 (complex) cells with respect to small type 1 (simple) cells was evident with age progression, reaching a minimum at 12 months (Figure 2B).

### DCX/PSA-NCAM and DCX/NeuN coexpression at different ages

The cytoskeletal protein DCX and the membrane-bound, anti-adhesive molecule PSA-NCAM are known to be generally coexpressed in cortical immature neurons (Rotheneichner et al., 2018; Bonfanti and Seki, 2021; Coviello et al., 2021), both being downregulated with maturation (Rotheneichner et al., 2018). On the other hand, NeuN an RNA-binding protein expressed by post-mitotic neurons that reached a high degree of differentiation (Mullen et al., 1992), can identify most types of mature neurons and is expressed by many type 2 cINs (Piumatti et al., 2018). On these bases, we explored whether such coexpression is stable in the mouse piriform cortex at different ages, by focusing on young (3 months old) and aged (15 months) animals (Figure 3). The analysis revealed coexpression of the two markers in immature cells of the piriform cortex, involving all morphological types: type 1 cells, type 2 cells and transitional forms (cells with soma size intermediate between type 1 and type 2 cells, and moderate dendritic ramification; Piumatti et al., 2018), at all the ages investigated (yellow arrowheads in Figure 3). In type 1 DCX^+^ cells, PSA-NCAM was present on both cell soma and processes, while in type 2 cells and transitional forms PSA-NCAM was distributed more heterogeneously (being present either on both the cell soma and process, only on the cell soma or only on processes, possibly reflecting different maturational stages). Occasionally, some type 2 DCX^+^ cells were devoid of PSA-NCAM, likely corresponding to more mature forms (having lost PSA-NCAM expression) (red arrowheads in Figure 3). Rare cells single-stained for PSA-NCAM, not expressing DCX were observed. These elements might be a scarce type of interneurons (Gómez-Climent et al., 2011; Figure 3, green arrowheads). The coexpression of DCX and PSA-NCAM was always consistent and strong in the newly generated cells of the neurogenic sites (Figure 3A, top), as previously described (La Rosa et al., 2019).

In DCX/NeuN double staining, coexpression was frequently observed in type 2 cells with mature morphologies, both at 3 and 15 months (Figure 3A, bottom). Type 2 cells expressing only DCX were also detected both at 3 and 15 months (red arrowhead in Figure 3A, bottom), likely reflecting more immature forms which will express NeuN later (e.g., transitional forms coexpressing PSA-NCAM in the correspondent double staining). Counting of DCX/PSA-NCAM and DCX/NeuN double staining are reported as pie charts and percentages in Figure 3A. While coexpression with PSA-NCAM remained substantially unvaried from 3 to 15 months of age, coexpression with NeuN increased with age, more than doubling at 15 months.

Since previous studies using transgenic reporter mouse suggested that some oligodendrocyte precursors may express DCX (Rotheneichner et al., 2018), we checked for possible coexpression of DCX and the Neuron Glial antigen 2 (NG2) by performing double staining at 3 and 15 months of age. However, in our system, we did not find any cells coexpressing DCX and NG2 across the piriform cortex, at young or at old age (Figure 3C). In addition, all cells single stained either for DCX or for NG2 displayed clearly distinct morphologies, typical of type 1 and type 2 immature neurons and multipolar oligodendrocyte progenitor cells, respectively (Figure 3C). Thereby, the entire population of DCX^+^ cells described and quantified in this study accounts for *bona fide* Type 1 and Type 2 cINs.

## Discussion

The recent identification of DCX^+^ cells in the piriform cortex layer II as non-newborn, “immature” or “dormant” neurons (history reviewed in Bonfanti and Seki, 2021), has introduced a possibility to place new, functional neurons in postnatal/adult neural circuits in the absence of stem cell division (Benedetti and Couillard-Despres, 2022), in parallel with the well-known process of stem cell-driven neurogenesis occurring in the brain neurogenic sites (SVZ and SGZ; Bond et al., 2015; Lim and Alvarez-Buylla, 2016; La Rosa et al., 2020a). Of interest, this “neurogenesis without division” does occur in the cerebral cortex, namely a region devoid of active stem cells/stem cell niches, which is of uttermost importance for high-order cognitive functions (Roth and Dicke, 2005), and known to be affected by many forms of dementia/neurodegenerative pathologies (Vinters, 2015). With these premises, questions arise about how these “young” neurons are temporally distributed across the lifespan and whether they can represent a sort of “reservoir” for the adult/aging brain (König et al., 2016; La Rosa et al., 2019). Or, alternatively, whether their occurrence might follow a progressive reduction, similarly to what observed for other forms of plasticity (Bonfanti, 2011; Paredes et al., 2016; Parolisi et al., 2018; Snyder, 2019; Bonfanti and Charvet, 2021). Here, by analyzing the number, morphology, and immunocytochemical features of cINs in the mouse piriform cortex from postnatal to old stages, we asked whether this cell population either undergoes a progressive decrease in number or remains stable, and how the pattern might be related to the well-known decrease of adult neurogenesis (Ben Abdallah et al., 2010).

### Occurrence of layer II cortical “immature” neurons progressively decreases across lifespan

**FIGURE 4:**
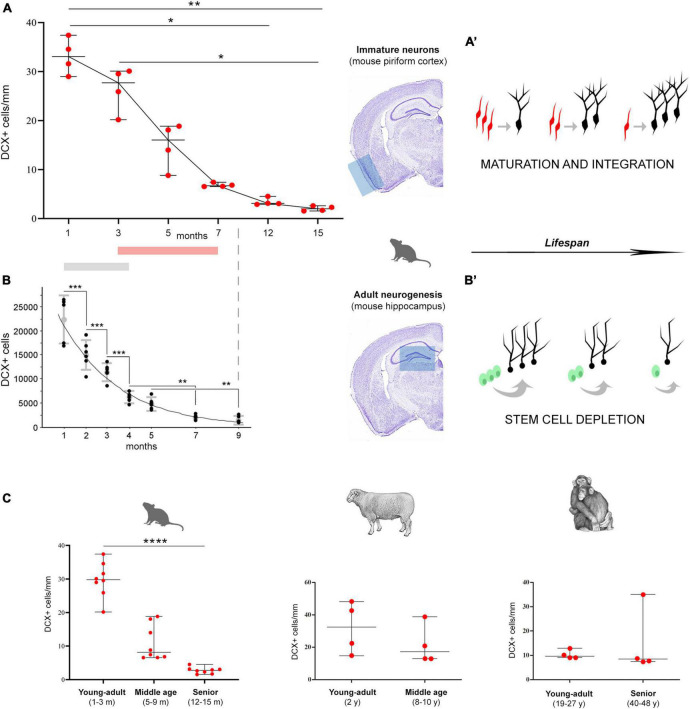
Age-related decrease of different populations of DCX^+^ cells in mice **(A,B)**, and in comparison with other mammals **(C)**. **(A,B)** Comparison between non-newly born, immature neurons of the piriform cortex [cINs; **(A)**] and newly born neurons of the hippocampal dentate gyrus **(B)**. **(A)** Data obtained in the present study demonstrating the decrease of cINs during life (*p < 0.05; **p < 0.01); **(B)** panel reproducing Figure 5 in Ben Abdallah et al. (2010; modified with permission from Elsevier), showing the decrease with age of DCX^+^ cell numbers in the mouse dentate gyrus (**p < 0.01; ***p < 0.0001). Gray and pink bars indicate the period in which the main drop occurs. **(A’,B’)** Schematic representation of the two distinct processes, both leading to integration of new functional elements (black neurons), either starting from cINs [red, in the cortex; **(A’)**] or from stem cell division [green, in the neurogenic sites; **(B’)**]. Both processes undergo progressive age-dependent reduction, yet, for different reasons. The apparent decrease of immature neurons (a reduction in the number of DCX^+^ cells) does correspond to a maintenance of the population through the maturing cells. On the other hand, in neurogenic processes the reduction really affects the production of new neurons, due to stem cell depletion/quiescence. **(C)** DCX^+^ immature neuron amount (cortical layer II linear density) in the paleocortex of three widely different mammals, at different ages (left; *n* = 8 for each life stage; ****p < 0.0001). Despite the substantial decrease in DCX^+^ immature neurons observed in rodents (left; *n* = 8 for each life stage), a substantial maintenance of these cells characterizes the large-brained, gyrencephalic mammals, such as sheep and chimpanzee [row data from La Rosa et al. (2020b); eLife Sciences Publ Ltd.; *n* = 4 for each life stage], thus suggesting a slowing down of the process with increasing brain size, lifespan, and higher-order cognitive abilities.

Our findings show that occurrence of DCX^+^ cINs is very high during juvenile stages, declining at young-adult ages to reach lower levels with aging (Figure 2). Since these cINs do not depend on stem cell activity (they are produced during embryogenesis and then persist as immature, “dormant” elements in postnatal and adult ages, Rotheneichner et al., 2018; Benedetti et al., 2020; reviewed in La Rosa et al., 2019; Benedetti and Couillard-Despres, 2022), their progressive reduction can be explained by slow maturation and subsequent integration into the layer II of the piriform cortex, occurring at different ages and involving the loss of DCX expression (Benedetti and Couillard-Despres, 2022). Hence, unlike neurogenic processes, which produce less neurons with increasing ages (Luo et al., 2006; Ben Abdallah et al., 2010; Encinas et al., 2011; Figure 4B), the “disappearing” cINs do persist as mature neurons, which are no more visible by DCX immunocytochemistry, due to their maturation (schematic representation in Figure 4A’). Such “apparent disappearance,” corresponding to maturation and integration, has been well demonstrated using a DCX-CreERT2/Flox-EGFP transgenic mouse, in which the immature cells can be visualized with green fluorescent protein, and followed through time after the loss of their DCX-expression (Zhang et al., 2010; Rotheneichner et al., 2018; Benedetti and Couillard-Despres, 2022). In this transgenic model, the vast majority of the cINs were found to accomplish their maturation through the animal life, the cells lost through aging being negligible (Rotheneichner et al., 2018). In other words, the apparent disappearance of the DCX^+^ cells in the piriform cortex observed at increasing ages, consists of an activation of previously inactive, “dormant” elements, thus maintaining the cell population. Here, we estimated the cell population of immature elements hosted in the piriform cortex in about 18,000 cells/hemisphere at 1 and 3 months of age, then progressively dropping with age, and leaving about 1,700 cells at 15 months (see Supplementary Tables 1, 2). These numbers represent respectively 3% and 0.3% of the total amount of neurons in the piriform cortex (considered to be around 500,000 in the three-month-old mouse; Srinivasan and Stevens, 2017), which can potentially act as a reserve/enhancer for cortical plasticity. The fact that the number of sections considered in the counting was not high (three sections/animal) may explain the variability observed between animals, what could represent a limit of this study. Yet, the entire ventral-to-dorsal extension of the piriform cortex was considered in each section, and significant decrease of the number of DCX^+^ cells was found at different ages, despite interindividual variability.

No big differences in the amount of cINs were observed by comparing the three anterior-posterior subregions of the piriform cortex at different ages, apart from a slight prevalence of DCX^+^ cells in middle and caudal regions with respect to the rostral one (Figures 1B, 2C”), indicating that the total number of cINs at each age and their age-related decrease can be considered a general feature of the piriform cortex. We did such a distinction since a recent study showed that piriform cortex connectivity is spatially structured in triadic circuit motifs along its anterior-posterior axis (Chen et al., 2022). Single olfactory bulb neurons targeting a particular location along the anterior-posterior axis of piriform cortex also project to matched, functionally distinct, extra-piriform targets (e.g., anterior olfactory nucleus, amygdala, or lateral entorhinal cortex), and neurons in the piriform cortex complete the triad, also projecting to the same extra-piriform target (Chen et al., 2022). Our results suggest that plasticity potentially involving the cINs of all these circuits undergo the same age-related changes across the lifespan.

The general decrease observed here for the cINs show similarities with that of hippocampal adult neurogenesis (Ben Abdallah et al., 2010; see Figures 4A, B and related section, below) and, to a lesser extent, olfactory bulb/subventricular zone (Luo et al., 2006), thus suggesting that all these forms of plasticity play important roles mostly at juvenile ages.

### Variation in cell types and marker coexpression linked to different maturational stages

The two cell morphologies indicated as type 1 and type 2 cells are known to represent the extremes of the maturational process of cINs during the period of DCX expression (Figures 1C, 4B’). Such maturation has been well characterized using the DCX-CreERT2/Flox-EGFP transgenic mouse, in which the immature, DCX-expressing cells can be followed across time with green fluorescent protein, until full maturation (and consequent loss of DCX staining) and functional integration into the layer II circuits (Rotheneichner et al., 2018; Benedetti et al., 2020). Of course, each brain section immunocytochemically stained with markers of maturity/immaturity (e.g., NeuN, DCX, PSA-NCAM) can include a gradient of maturational forms between the two extremes (see Figures 1, 3B). In the temporal window of DCX expression, the small, unipolar-bipolar type 1 elements are the most immature DCX^+^ neuronal precursors that, after progressive maturation increase their soma size and grow a ramified dendritic arborization, becoming the type 2 “complex cells” (Piumatti et al., 2018; Rotheneichner et al., 2018; La Rosa et al., 2020b; Benedetti and Couillard-Despres, 2022; Figure 3B). Here, we counted the cells falling into the category of type 1 and type 2 morphologies and their ratio was analyzed at each age (Figure 2B). As expected (see Piumatti et al., 2018; La Rosa et al., 2020b), type 1 cells were prevalent at all the ages, yet the percentage of type 2 cells was decreasing with age (from 1 to 12 months), with a small increase at 15 months (Figure 2B). This pattern, along with that reported above for total DCX^+^ cells, suggests that a small amount of highly immature (type 1) cells might represent a reservoir for plasticity in the adult/aging brain.

Two additional markers were used in association with DCX to assess the neuronal maturational stage of the cINs, as described in Piumatti et al. (2018) and Coviello et al. (2021) (Figure 3): PSA-NCAM, a low-adhesive form of N-CAM widely present in neurons during brain development and expressed by cells retaining plasticity during adulthood (Hoffman and Edelman, 1983; Bonfanti, 2006; Bonfanti and Nacher, 2012); NeuN, expressed by post-mitotic neurons starting differentiation (Mullen et al., 1992), which can identify most types of mature neurons (Gusel’nikova and Korzhevskiy, 2015), and is expressed in type 2 cINs [Piumatti et al., 2018; referred to as “complex cells” in Rotheneichner et al. (2018)].

The analysis carried out on DCX/PSA-NCAM double staining revealed coexpression of the two markers in most immature cells of the piriform cortex, involving all morphological types: type 1 cells, type 2 cells and transitional forms, at all the ages investigated (Figure 3B). Only few DCX^+^ type 2 cells were devoid of PSA-NCAM, likely corresponding to the most complex forms, close to the accomplishment of their full maturation (Figure 3B). Accordingly, it was recently demonstrated that PSA-NCAM depletion promotes maturation of cINs in rodent paleocortex (Coviello et al., 2021), thus indicating that loss of PSA-NCAM can precede loss of DCX. Concerning the heterogeneous coexpression of PSA-NCAM and DCX in cell soma and/or cell processes, the heterogeneity may therefore reflect different stages of maturation. Counting of the DCX/PSA-NCAM double-stained cells at 3 and 15 months of age revealed substantially similar values of coexpression (pie charts in Figure 3A), indicating that a population of highly plastic neurons is maintained through ages.

On the other hand, the increase in the number of DCX^+^ cells coexpressing NeuN (more than doubling at 15 months; pie charts in Figure 3A) might be caused by a slowing down of the maturational process with increasing age, resulting in some cells being “blocked” in a “late immature” state. Under such assumption, one could also explain the decrease of type 2 cells with respect to type 1 cells in older animals. Namely, the age-related decrease in the percentage of type 2 cells would be justified by a dwindling, yet maintained, population of ever-immature type 1 cell (small reservoir of highly plastic cells), on one hand, and by some type 2 cells slowing down their maturation with age, on the other.

### Comparison with adult hippocampal neurogenesis

The findings obtained in the present study on the cIN age-related variation in the piriform cortex appear quite similar to those reported for the neurogenic process of the hippocampal dentate gyrus in C57BL/6 mice from 1 to 9 months (Ben Abdallah et al., 2010), since both processes undergo substantial reduction with increasing age (Figures 4A, B). It is clear that cINs and hippocampal adult neurogenesis are different processes, which are not fully comparable; nevertheless, they both represent forms of neurogenesis, in terms of addition of new neurons. Moreover, they both show a progressive exhaustion of available cell types: of cells retaining immaturity in the former, and of actively dividing stem cells and their product in the latter (Figures 4A’, B’). Here we focus on the time period extension in which they are still detectable, because they show interesting differences beside a common trend of age-related decline. Our results reveal a strong decrease of cINs between 5 and 7-months, while data from Ben Abdallah et al. (2010) in the hippocampus show this decrease already starting from 1–3 months (Figures 4A, B; Table 1). In addition, by considering the percentage of decrease in DCX^+^ cells from their initial occurrence at 1 month to subsequent ages, it is only 19% in the piriform cortex against 97% in the hippocampus at 3 months, and 394% against 1,094%, respectively, at 7 months. In the piriform cortex of older animals, cINs reach a value comparable to that of the hippocampus at 9 months, only at 15 months (Table 1), indicating a longer persistence of cINs with respect to cells involved in adult neurogenesis. These data suggest that exhaustion of the cIN reservoir does occur in a more diluted way with respect to adult neurogenesis. As a matter of fact, in the 15-month-old mouse piriform cortex there are still 1,680 DCX^+^ cells, namely a similar or higher amount with respect to those present in the hippocampus 6 months before (about 1,300 at 9 months; Ben Abdallah et al., 2010).

Of course, our data and data collected from Ben Abdallah are heterogeneous. First of all, our quantification of DCX^+^ cells was performed using a direct cell counting on ImageJ software instead of stereological methods with Stereoinvestigator, as done by Ben Abdallah et al. (2010). We chose this method because of the low number of cINs: counting with stereological methods are recommended when a considerably larger number of particles per individual are present (700–1,000; Herculano-Houzel et al., 2015). Moreover, the ages considered in these two studies are only partially overlapping (Figures 4A, B): differences in cIN abundance is only significant when comparing 1-month-old to 12- and 15-months-old mice, hence no closer ages (e.g., 2, 4, 9 months) were considered; on the other side, hippocampal adult neurogenesis highlights significant differences starting from the first months of life, requiring the analysis of closer ages.

Overall, the decrease of cINs during young/adult stages can fit with the general view of structural plasticity as a process which is prevalent during youth, to allow the refinement of brain circuits on the basis of experience (in mouse, mainly linked to olfactory experience). Yet, a more moderate decrease of cINs with respect to adult neurogenesis, can leave a “small reserve” of young elements even during adult/senior stages. A fact that seems even more evident in large-brained mammals (see below).

### Comparison with data available in large-brained mammals and humans: a link with lifespan?

Comparative studies carried out in mammals revealed the existence of remarkable interspecies differences in the occurrence, distribution, and amount of cINs, with greater abundance of these cells in large-brained, gyrencephalic mammals (Piumatti et al., 2018; La Rosa et al., 2020b), including humans (Li et al., 2023). In addition, another difference seems to be linked to lifespan: in large-brained, long-living species the cINs do persist at adult/old stages, not showing the same decrease observed in mice (Figure 4C). In chimpanzees of young-adult and senior ages (19–27 and 40–48 years old, respectively; La Rosa et al., 2020b), as well as in sheep of young-adult and middle ages (2 and 8–10 years old, respectively; La Rosa et al., 2020b), no substantial reduction was observed in the amount of cINs within the piriform cortex (Figure 4C) and moderate reduction was seen in the neocortex (La Rosa et al., 2020b). A possible trend of “stabilization” in the amount of cINs in large-brained, long-living species, is supported by the highly similar pattern obtained by comparing young-adult with middle age stages in sheep, with young-adult and senior stages in chimpanzee (Figure 4C), indicating a long-lasting maintenance of the DCX^+^ cell population in mammals with longer lifespan. In humans, no comparable quantification is available at present, yet the cINs can be observed in aged individuals, being mostly maintained in the temporal lobe (Coviello et al., 2022, age range: 5–62 years; Li et al., 2023, age range: 6 months–100 years). An even higher stabilization through age has been observed for DCX^+^ neurons in subcortical regions (Piumatti et al., 2018).

Overall, the currently available results indicate that mammalian species characterized by small, lissencephalic brain, short lifespan and rapid development of the nervous system (such as the laboratory mouse) are endowed with a small population of cINs, which is restricted to the paleocortex and follows a temporal pattern of reduction very similar to that of stem cell-driven adult neurogenesis. By contrast, large-brained, long-living species, which have far higher amount and more widespread distribution of cINs (including the entire neocortex) seem to maintain longer these populations of “young” neurons across adulthood and aging, suggesting a slowing down of their maturation/integration through the lifespan. The idea that cINs might represent a “cognitive reserve” for the cerebral cortex in long-living species (Palazzo et al., 2018; La Rosa et al., 2019) remains a stimulating hypothesis, nevertheless further studies systematically considering different ages in primates and humans are needed.

## Conclusion

Recent findings revealed that brain structural plasticity can occur in different forms varying with age, brain region, and species, thus stressing the importance of defining plasticity along temporal and spatial dimensions (Bonfanti and Charvet, 2021). Stem cell-driven adult neurogenesis is a developmental process particularly useful during juvenile stages of the animal lifespan (Ben Abdallah et al., 2010; Sanai et al., 2011; Sorrells et al., 2018; Snyder, 2019; Bond et al., 2022). It supports the postnatal growth of the brain and the refinement of its neural circuits based on experience (Semënov, 2019; Seki, 2020; Kempermann et al., 2022; Bogado Lopes et al., 2023), but undergoes a progressive depletion of neural stem cells and/or entry in a quiescent state during adulthood (Encinas et al., 2011; Urbán et al., 2019). As such, it is different from the continuous cell renewal maintaining homeostasis in other stem cell systems of the body through the entire lifespan (Semënov, 2019).

Findings of the present study, along with recent comparative data obtained in large-brained, long-living mammals, do suggest that cINs can behave conversely, by maintaining their presence when the stem cell-driven neurogenesis has started to decline. In this process, interspecies differences are important: stem cell-driven neurogenesis and cINs appear to behave similarly in rodents but differently in large-brained species, suggesting a positive selection of cINs across evolution, as a preferential form of plasticity in large-brained mammals (Palazzo et al., 2018; La Rosa et al., 2020b; Ghibaudi and Bonfanti, 2022). Their independence from stem cell division can be an advantage, likely at the basis of the evolutionary choices that supported their widespread occurrence in the neocortex. Furthermore, in longevous species there might have been a selective pressure to develop a pool of long-lasting cINs in the expanded cortical areas, while the same pressure did not apply for the neurogenic niche pool.

In conclusion, considering the temporal dimension of plasticity in mammals, it is possible that the situation of stem cell-driven neurogenesis in young-adult rodents is more similar to human childhood (Bond et al., 2022), whereas for cortical immature neurons the process might be extended/diluted in time (La Rosa et al., 2019, 2020b; Li et al., 2023), providing a “small reserve of young neurons” even at advanced ages.

## Data availability statement

The raw data supporting the conclusions of this article will be made available by the authors, without undue reservation.

## Ethics statement

The animal study was reviewed and approved by “Directive 2010/63/EU of the European Parliament and of the Council of 22 September 2010 on the protection of animals used for scientific purposes,” according to protocol number 785_2019PR, following the Institutional guidelines of IBBC/CNR and the approval of the Ethical Committee. Mice C57/BL6 (Charles River Laboratories, RRID:MGI:3696370).

## Author contributions

LB and SF-V conceived and designed the work. LB, MG, and SF-V wrote the manuscript. SC-D, BB, and CL edited the manuscript. LB, MG, SC-D, BB, and SF-V provided conceptual feedback. CL performed statistical analyses. SF-V and SC-D provided animals. EV, MG, and NM performed the analysis. LB, SF-V, SC-D, and BB provided funding. All authors contributed to the article and approved the submitted version.
