# Supplementary material for: Age-related changes in layer II immature neurons of the murine piriform cortex
Source: Front Cell Neurosci. 2023 Jul 28;17:1205173. doi: 10.3389/fncel.2023.1205173 (PMC10416627; doi:10.3389/fncel.2023.1205173)
Supplement: Supplementary file 1 [file Data_Sheet_1.docx]

Supplementary Material

**Age-related changes in layer II immature neurons of the murine piriform cortex**

Marco Ghibaudi^1,2#^, Nicole Marchetti^3#^, Elena Vergnano^1^, Chiara La Rosa^1^, Bruno Benedetti^4,5,6^, Sebastien Couillard-Despres^4,5,6^, Stefano Farioli-Vecchioli^3^*, Luca Bonfanti^1,2^*

*** Correspondence:**

Luca Bonfanti, DVM, PhD

Department of Veterinary Sciences

Largo Braccini 2, 10095 Grugliasco (TO)

University of Turin, Italy

Email: [luca.bonfanti@unito.it](mailto:luca.bonfanti@unito.it)

Stefano Farioli-Vecchioli, PhD

Institute of Biochemistry and Cell Biology

National Research Council

Via E. Ramarini 32, Monterotondo, 00015 Rome, Italy

Email: [ste](mailto:stefarioli@hotmail.com)fano.fariolivecchioli@cnr.it

**Supplementary Figures and Tables**

**Supplementary Figures**


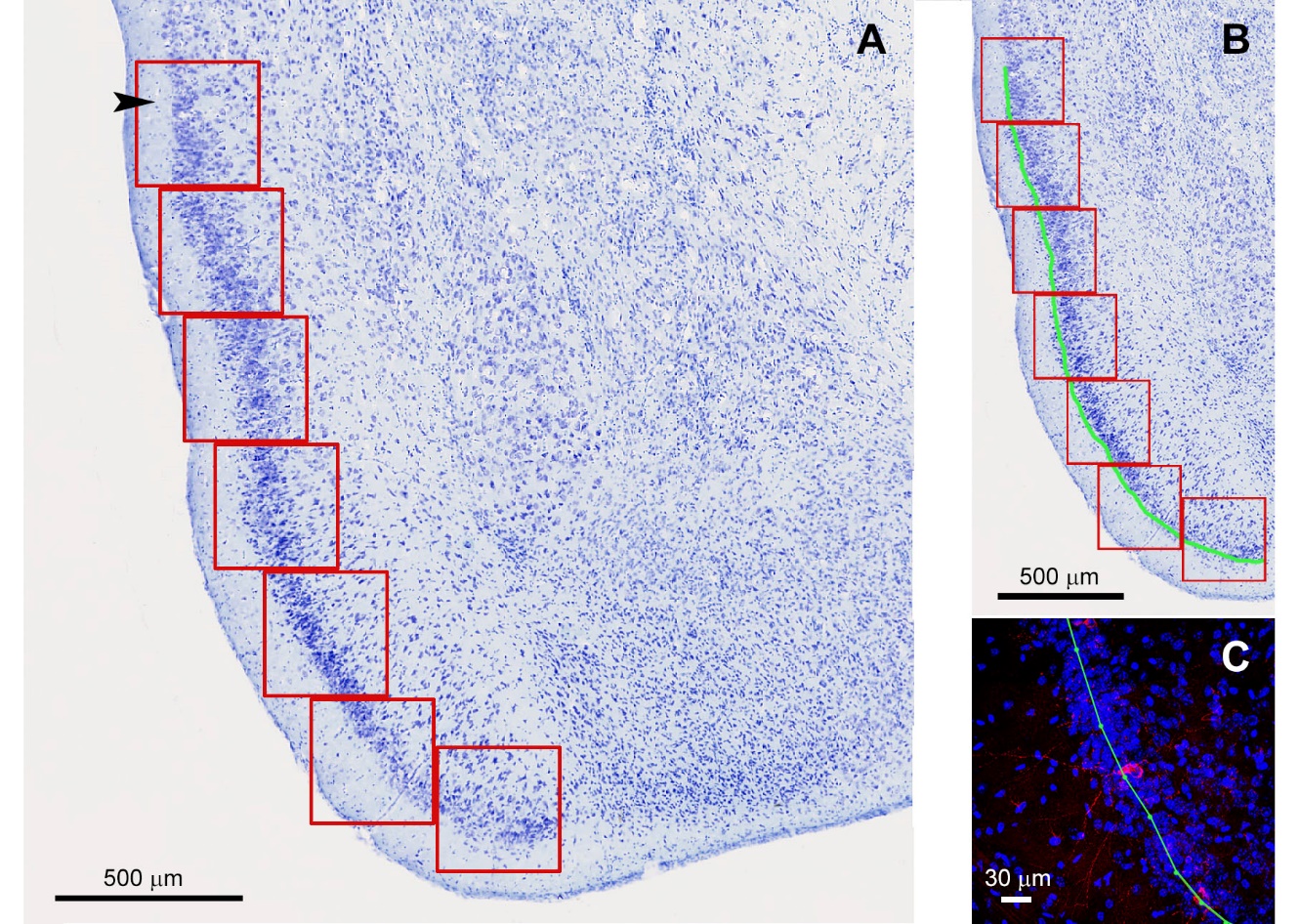


**Supplementary Figure 1.** Counting method in each coronal section of the piriform cortex (at anterior, middle, and posterior sites). (A) Adjacent confocal fields (318,215 x 318,215 µm wide; here represented in red on a toluidine blue-stained section of the middle piriform cortex at 7 months of age) were used to cover the entire ventral-dorsal extension of the piriform cortex (allocortex, but also recognizable at the confocal microscope by the high density of DAPI+ nuclei).

(B) The total length (ventral-to-dorsal) of the piriform cortex in each section was obtained by tracing the length of cortical layer II in each confocal field on imageJ (green line, C). Arrowhead: allocortex/isocortex transition.

**Supplementary Tables**

**Table S1. Estimation of total piriform cortex length (anterior-to-posterior) and total DCX+ cells in the piriform cortex (one hemisphere)**

| **Age** | **Piriform cortex coronal sections in a whole hemisphere** | **Section thickness** | **Total piriform cortex length (anterior-to-posterior)** | **Total DCX+ cells/section**  **(mean from rostral, middle, and caudal sections)** | **Estimation of total DCX+ cells in piriform cortex (one hemisphere)** |
| --- | --- | --- | --- | --- | --- |
| 1m | **120** | **40** **µm** | **4,8** **mm**  (120x40: 4800 µm) | **151,75** | **18.210** (151,75x120) |
| 3m |  |  |  | **152,25** | **18.270** (152,25x120) |
| 5m |  |  |  | **90,25** | **10.830** (90,25x120) |
| 7m |  |  |  | **37,5** | **4.500** (37,5x120) |
| 12m |  |  |  | **23,5** | **2.820** (23,5x120) |
| 15m |  |  |  | **14** | **1.680** (14x120) |

**Table S2. Percentage of DCX+ cells over piriform cortex whole neuronal cell population**

| **Age** | **Estimation of total DCX+ cells (see Table S1)** | **Estimation of neuronal number in the three month-old mouse piriform cortex** (Srinivasan & Steven, 2017) | **% of DCX+ cells** |
| --- | --- | --- | --- |
| 1m | 18.210 | 532.617 cells | 3% |
| 3m | 18.270 |  | 3% |
| 5m | 10.830 |  | 2% |
| 7m | 4.500 |  | 1% |
| 12m | 2.820 |  | 0,5% |
| 15m | 1.680 |  | 0,3% |

**Supplementary Data**

**Counting of DCX+ cells in the piriform cortex of each animal and ages (N=4 mice/age), including differential counting of type 1 and 2 cells**

See the Excel file at the link to Supplementary material
